# Identifying children at risk of malnutrition

**DOI:** 10.1186/s12937-018-0392-4

**Published:** 2018-09-15

**Authors:** Alan A. Jackson

**Affiliations:** Emeritus Professor of Human Nutrition, University of Southampton, Southampton General Hospital (MP 113), Tremona Road, Southampton, SO16 6YD UK

This issue of Nutrition Journal includes three papers by Grellety and Golden, which explore comparisons between the use of mid-upper arm circumference (MUAC) and the use of weight in relation to height (WH) to screen for and identify children with severe acute malnutrition at risk of death [1–3]. This important debate is the most recent in a long history of attempts to understand the nature of a condition of varied geographical distribution with a complex aetiopathology in which the risk of mortality is high and effective care needs can appear counterintuitive. The condition is common where resources are limited and hence identification and effective care requires simple approaches that can be delivered at community level, but more complex problems need to be securely identified and manged in a facility [4, 5]. Grellety and Golden [1–3] present data that suggest that the current balance of effort allows unacceptable mortality because groups of children at greatest risk based upon WH or the presence of oedema are not adequately identified when using MUAC, and hence, not offered appropriate care. If the authors are correct, the problem needs to be acknowledged in order for better approaches to be considered and put in place.

One of the great marks of the progress of society over the past 50 years has been the considerable improvements in the life opportunity for children, marked as significant reductions in post-neonatal mortality for children under 5 years of age [6]. A wide range of players can be credited with making contributions to these singular achievements [4–6]: contributions which embrace a rights-based approach to health and consideration of social factors and also the biomedical interventions required to save life and enable normal development in children at risk [4, 5, 7, 8]. One of the major challenges in efficiently facilitating progress has been the extent to which the problem of malnutrition is conceived of as a social problem or a medical problem. The reality is of course that social progress itself is often manifest as changing patterns of ill-health, which present as medical problems [4, 5, 9].

Given that malnutrition is prevalent in many widely different contexts [4, 5, 10], its genesis and any approach to its alleviation has always excited strong differences of opinion about how it might best be tackled. The priority given to any of the widely different approaches that might be adopted has often depended upon the particular interests and direction of concern of those immediately involved. The model articulated by UNICEF of immediate, underlying, and basic causes acknowledges multiple levels of concern, each of which has to be the focus and responsibility of different groups, but each of which has implications for the other levels; therefore, all have to be addressed with some measure of balance [11, 12]. At times, differences among approaches have evoked fierce controversies that have challenged our scientific insights, intellectual understanding, and our ability to translate theory into practice. These uncertainties have been particularly evident when the problems being addressed appear intractable, and hence, there is uncertainty about how best to proceed [7]. Further insights based upon advancement in technology, its application to health care, and the understanding generated from the available data have enabled resolution of differences, thereby clarifying the most appropriate approaches that not only embrace differences in viewpoints but draw strength from their resolution. At its heart, resolution of these uncertainties and differences reflects the importance of research and its application to the delivery of improved health care [4, 6, 8]. For example, the development of ready-to-use therapeutic foods derived from milk-based products for managing severely malnourished children has been informed by results of physiological and metabolic studies carried out in hospital, but has enabled a high standard of care in the community [13–16]. By applying the same principles, the further development of products based upon locally-available foods have made possible backward food production and employment opportunities and a move to local sustainability [17, 18]. The important underlying principle is that the nutrient composition of the therapeutic food seeks to correct common specific nutrient deficiencies that are not readily achieved through usual food mixes [13–16, 18, 19].

The characterisation and understanding of the pathophysiology of severe malnutrition has enabled the delivery of better services, both for its care and for its prevention [5]. At times, contention about the most appropriate approach to adopt has been vitriolic amongst strong personalities with passionate enthusiasm to improve outcomes. Williams [20, 21] and Waterlow [22, 23] both had experience characterising and treating the problem in West Africa and the Caribbean. Williams can be credited with the careful clinical characterisation of the kwashiorkor syndrome, its differentiation from marasmus, and its diagnosis, progress and treatment in the Gold Coast, now Ghana [20, 21]. This enabled others to better structure their clinical approach to care. As a physiologist clinician, Waterlow drew on the experience of Williams to develop a more secure framework for scientific investigation [4, 5, 23]. His group showed how modulation of physiology to achieve reductive adaptation enabled survival of the most nutritionally challenged, albeit at the risk of decreased resilience to metabolic imbalance or environmental stresses such as infection or emotional trauma. Applying this understanding to achieve successful care required carefully structured approaches, as exemplified in the WHO 10 steps [24], which in many regards were counterintuitive, especially for the sickest children [5, 8]. Central to this success has been the ability to recognise the imperative for correcting cellular functionality in order to improve appetite by correcting deficiencies of nutrients, such as potassium or zinc while limiting the therapeutic use of iron, before attempting to address the correction of a deficit in body composition as marked by anthropometry. The rebalancing of emphasis on the relative importance of energy and protein intake at different phases of treatment was critical to this changed perception, as was recognition of the need to correct specific deficiencies and deficits for a range of nutrients [5, 8]. Scrimshaw drew attention to the critical role played by the stress of infection and how this might increase losses of specific nutrients, such as potassium, phosphorus or zinc, and hence enhanced nutritional vulnerability, thereby enabling the condition to run an aggressively more lethal course in those already malnourished [4, 5, 25]. Ultimately, both the nurturing viewpoint emphasised by Williams and the harder scientific perspective favoured by Waterlow feature in appropriate care and are reflected in the WHO 10 point guidelines for the care of children with severe acute malnutrition [23].

During the 1960s and 1970s, the debate around whether or not it was best to manage children in hospital or in the community raged, but the importance of being able to identify children at risk and direct them to appropriate care was never in dispute [26]. Given restricted access to hospitals, Morley placed justifiable emphasis on approaches that could operate at the community level by “doing simple things well”. By developing effective screening of children using assessment in the community, he showed how worthwhile progress could be made [27]. At the same time, placing emphasis on greater household food security had an important part to play. Sen placed emphasis on the important social and economic implications of ensuring food availability, but also drew attention to the importance of food distribution, with appropriate attention being given to both food quality and food quantity [9].

Thus, community based care is critically dependent upon the ability to identify those who are already sick, but also has to pay special attention to those who are particularly vulnerable and at high risk of mortality. Abnormal anthropometry marks the result of a period of poor food intake. When carried out well, the use and application of anthropometry can play an invaluable role in ordering the delivery of services in such cases. A conceptually simple study carried out in Jamaica showed that the use of regular anthropometry in young children made it possible to identify and manage those already malnourished, as well as identify those at increased risk, and offer care to prevent further deterioration with clear benefit in the first year and sustained benefit over the next three years. Together, this combined approach substantially reduced the vulnerability of this population [28, 29]. At the time, these investigators noted that different children were identified when their anthropometric risk was based upon MUAC compared with when it was based upon weight for height [29]. It has remained an open question as to what this difference represented, and what might be the implications for care and outcome.

By 1981, the WHO were able to offer guidelines for the treatment of severe malnutrition that, when followed, could result in substantial reductions in mortality. However, in practice, the advice was difficult to follow and required skills and capabilities that are not usually found in clinical paediatric services nor in clinics where malnourished children most usually present, if they were present at all [29]. The practicalities of identifying children at risk through screening with the use of MUAC and the ready availability of effective therapeutic foods, first developed for practical use in emergencies, changed this landscape completely [8, 13–16]. For the first time, the integrated management of severe acute malnutrition was made practical, by ensuring a more secure diagnosis associated with an effective therapeutic intervention [15, 16]. The sickest children with severe malnutrition and complications are most effectively managed in clinical facilities. The greater proportion of children who have severe acute malnutrition are without complications and are better managed under supervision in an outpatient clinic environment. Those identified as affected by moderate malnutrition are still at some risk, but are less vulnerable and can be securely managed in a well organised community outreach programme [30, 31]. The enormous progress in developing these capabilities and their application in practice is a great credit to those thought leaders, scientists, and practitioners who have ensured that a problem that at one time seemed intractable now comes within the grasp of manageable.

Every success throws up another series of problems and the three papers in this issue, by Grellety and Golden, identify and explore one such concern. As alluded to above, there is evidence from a wide range of populations showing that those identified as being severely malnourished based upon MUAC as the anthropometric indicator are often different from those identified as being severely malnourished based on weight for height [32]. In other words, the two indicators do not necessarily identify the same children. This is of considerable importance for the delivery of services. There are choices that have to be made and pragmatically, the simplest approach that gives a workable solution is usually preferable [15, 16]. It is based on this premise that the adoption and use of MUAC has enabled effective care to be brought to many vulnerable children and in that way, saved many lives. The more challenging assessment and quality assurance based upon the determination of weight for height requires more complex instrumentation with an associated need for maintenance, together with a skill and capability from staff that is challenging in many situations. When direct comparisons have been drawn between the two measures in identifying risk, the use of MUAC has appeared to be the most reliable [10]. However, over the recent past, as the delivery of improved care has been more widely available in many more centres, and with better quality measurements, concerns have been raised. In practice, health practitioners, particularly those working in hospital-based facilities, have had an increasing concern that they might miss the identification of the need for special care in children who appear vulnerable based upon a measurement of weight for height, but who have not been identified as being at risk based upon MUAC. Thus, there has been concern and debate around proposals to use MUAC as the sole screening tool.

Grellety and Golden have been active participants on one side of this debate. On the other has been a group of highly regarded clinicians with considerable experience in the identification and care of malnourished children, especially in the community. Both sides have made substantial contributions to our understanding and the practical delivery of effective care in the most difficult of circumstances. The experience and insights offered by both sides are to be respected. The three papers in this issue from Grellety and Golden offer their most recent perspective on the problem [1–3]. They have sought to bring together a considerable literature that captures the experience of using the different measures to identify and diagnose the different phenotypes embraced within the term severe acute malnutrition, and the mortality related to each of the different clinical syndromes of severe acute malnutrition: kwashiorkor, marasmus, and marasmic-kwashiorkor. Their analysis suggests that earlier interpretations of the available data have within them statistical insecurities that in their adoption and application have led to unacceptable mortality in children. This is because by only assessing children on the basis of MUAC, there would have been a failure to identify and treat children who would have been identified as vulnerable on the basis of weight for height. As they state, if this insecure interpretation of the risks involved is not addressed, it can have serious implications that should be taken into account in terms of policy and practice.

Further, these observations and their interpretation sets a great challenge to the scientific and clinical community to revisit our understanding of the relationship between measures of body composition and the changes in pathophysiological function that relate directly to adverse risk for the different malnutrition phenotypes [4, 5]. There is the need to relate molecular, cellular and tissue changes, alterations in inflammatory and immune competence, and the nature of the inter-relationship with a modified microbiome to those anthropometric or other measures of body composition that have been used to characterise risk. How these relate to actual risk and respond to different care options marks a new agenda for nutrition research and child health. Modern investigative approaches, if properly deployed, offer tremendous opportunity for making progress in this regard. The knowledge and understanding that could come from work of this kind would provide a refinement of our appreciation of human biology that would contribute directly to a better understanding of the nature of the double burden of malnutrition. This has special resonance for the current epidemic of non-communicable disease that is sweeping the globe and is most readily marked by alterations in body composition, conveniently characterised as obesity. There is a relationship between the factors that determine the pattern of tissue mobilisation and weight loss during the development of malnutrition and the factors that enable the pattern of nutrient partitioning and tissue deposition during recovery from a period of challenged nutrition. The determinants of the absolute amounts and relative proportions for the net deposition for length/height, lean mass, and fat mass are inadequately understood at all ages and stages of life. Their elucidation is central to the challenge for modern nutritional science and, in fact, represents a major scientific uncertainty of direct importance for ill-health and its management across all populations [7, 12].

Structuring the debate around these considerations and using the information that has been generated during the treatment of severe childhood malnutrition is of immediate practical value, but also provides an important and critical bridge between the nutritional science of the twentieth century and that needed for the twenty first century.
